# Balancing act: the complex role of NK cells in immune regulation

**DOI:** 10.3389/fimmu.2023.1275028

**Published:** 2023-11-02

**Authors:** Hongwei Jiang, Jingting Jiang

**Affiliations:** ^1^ Department of Tumor Biological Treatment, The Third Affiliated Hospital of Soochow University, Changzhou, Jiangsu, China; ^2^ Jiangsu Engineering Research Center for Tumor Immunotherapy, The Third Affiliated Hospital of Soochow University, Changzhou, Jiangsu, China; ^3^ Institute for Cell Therapy, Soochow University, Changzhou, Jiangsu, China

**Keywords:** NK cell, adaptive immunity, regulation, T cell, B cell, virus infection, tumor

## Abstract

Natural killer (NK) cells, as fundamental components of innate immunity, can quickly react to abnormalities within the body. In-depth research has revealed that NK cells possess regulatory functions not only in innate immunity but also in adaptive immunity under various conditions. Multiple aspects of the adaptive immune process are regulated through NK cells. In our review, we have integrated multiple studies to illuminate the regulatory function of NK cells in regulating B cell and T cell responses during adaptive immune processes, focusing on aspects including viral infections and the tumor microenvironment (TME). These insights provide us with many new understandings on how NK cells regulate different phases of the adaptive immune response.

## 1 Introduction

### 1.1 The development and maturation of NK cells

Derived from common lymphoid progenitor cells (CLP), NK cells serve as a vital element of the innate immune system. Both human and mouse NK cell development initiates from oligopotent CLPs. In mice, once CLPs are generated from multipotent self-renewing hematopoietic stem cells (HSCs), their subsequent differentiation pathways include NK cell progenitors (NKPs) (1). The definition of NKP cells is characterized by the expression of IL2RB (CD122) when CLPs enter the lymphoid lineage (2). The developmental process thereafter mainly consists of immature NK (iNK) and mature NK (mNK) stages.

The iNK stage begins with the expression of the NKG2D-DAP10 activation receptor complex by NK cells. During this stage, NK cells express NCR1, L-selectin (CD62L), DNAM-1 (CD226), NK1.1,NKG2A, and cell adhesion molecules. The expression of CD49b (DX5) and CD51 marks the transition of NK cells into the mature stage. The expression of different combinations of Ly49 receptors signifies the diversity of NK cell functions (3).

In contrast to mouse NK cells, which primarily mature in the bone marrow(BM), human NK cell development and maturation occur in both the BM and secondary lymphoid organs (4). Human NK cell development is categorized into six stages, with the transition from iNK to mNK status determined by the expression of CD56 (NCAM) (3). CD56^Bright^ NK cells are considered an early stage of maturation, while CD56^Dim^ NK cells are regarded as fully mature NK cells. CD56^Dim^ NK cells exhibit high cytotoxic activity and are mainly found in peripheral blood, where they efficiently kill target cells (5, 6). CD56^Bright^ NK cells have lower cytotoxicity but produce high levels of cytokines like IFN-γ and are commonly found in secondary lymphoid organs (7, 8).

After expressing Killer cell Lectin-like Receptor G1 (KLRG1), mNK cells partially migrate to secondary lymphoid organs (9, 10). Once NK cells reach maturity, they are extensively distributed throughout the body, including the BM, lungs, spleen, liver, lymph nodes(LN) and peripheral blood (11–14).

### 1.2 Activation and inhibitory receptors of NK cells

The activation state of NK cells is determined by the stimulation received through either activating or inhibitory receptors. When the signaling from activating receptors predominates, NK cells become activated; conversely, their activity is suppressed (15). Several previous reviews have provided comprehensive descriptions of NK cells’ activating and inhibitory receptors, encompassing their respective ligands and associated signaling molecules (16–18).

In general, these receptors can be categorized into the following families: Ly49, KIRs (Killer Cell Immunoglobulin-like Receptors), CD94-NKG2, NKG2D, and NCRs (Natural Cytotoxicity Receptors) (18). The Ly49 receptor family in mice shares similarities with the KIR receptor family in humans, although they do not have a one-to-one correspondence (19). Among the Ly49 receptors, Ly49D and Ly49H are activating receptors, while the rest are inhibitory receptors. Notably, the ligand for Ly49H is m157 protein, while the ligands for other Ly49 receptors are H-2D or H-2K molecules (18). Similarly, the ligands for KIR receptors are HLA molecules, and different receptors correspond to different ligands, including HLA-G, HLA-C, HLA-B, or HLA-A. Among them, KIR2DL1, KIR2DL2/3, KIR2DL5, KIR3DL1, and KIR3DL2 are inhibitory receptors, while others are activating receptors (17). The CD94-NKG2 receptor family is expressed on not only mouse but also human NK cells. NKG2A is an inhibitory receptor, whereas NKG2E and NKG2C are activating receptors. However, the ligands for these CD94-NKG2 receptors differ between mice (Qa1b) and humans (HLA-E) (18). NKG2D does not belong to the CD94-NKG2 family because it lacks the corresponding CD94 subunit to associate with (20). NKG2D is an activating receptor, with its ligands being ULBP1-4 and MICA/B in humans and H60, MULT-1, and RAE-1 in mice (17). The NCRs family mainly includes NKp46, NKp44, NKp30, etc. These receptors are all activating receptors for NK cells. NKp44 and NKp46 share the common ligand Viral HA, with NKp46 also sharing the ligand HSPG with NKp30. However, NKp30 has two additional ligands, BAT-3 and B7-H6 (18).

In addition to the receptor-ligand pairs described above, interactions such as 2B4 with CD48 (21), DNAM-1 with PVR (CD122) (22, 23), and LILR with MHC class I molecules (HLA class I molecules) (24) also significantly influence the modulation of NK cell states.

### 1.3 The role of NK cells in maintaining homeostasis in the body

In summary, NK cells play four primary roles in maintaining homeostasis in the body, including immune regulation, immune homeostasis, immune defense, and immune surveillance. Immune defense is the action of NK cells to resist foreign substances, encompassing their resistance to viruses, bacteria, and parasites (25). Immune surveillance refers to the process through which NK cells identify and eliminate aberrant cells within the body, where clearing tumor cells is the most common manifestation of their immune surveillance function (26). Tumor cells trigger NK cell cytotoxicity because they lack all or part of the MHC class I molecules, thus making them recognizable by NK cells as non-self cells and subject to elimination (27, 28). It is worth noting that in most conditions, NK cells mediate broad-spectrum cytotoxicity without the need for prior antigen stimulation (29), exhibiting non-specific and MHC-unrestricted cytotoxicity (30). However, recent research has provided evidence suggesting that peptide-specific recognition of HLA-I molecules is not confined solely to T cell receptors (TCRs) alone, KIRs on NK cells also exhibit a notable degree of specificity for HLA class I-peptide complexes. Therefore, the response of NK cells to infection or disease can also vary based on the immunopeptides bound to HLA-I molecules (31).

The immune homeostasis function exerted by NK cells primarily focuses on maintaining internal equilibrium and stability within the body. An essential manifestation of NK cells in self-regulation is their clearance of senescent cells, a process that requires collaboration with macrophages (32). NK cells recognize senescent cells through the NKG2D receptor and kill them through a perforin-dependent mechanism (33). Simultaneously, NK cells release cytokines to activate macrophages, which subsequently clear these senescent cells (32). The immune regulation function is another crucial role of NK cells that should be emphasized. Abundant cytokines such as IFN-γ, GM-CSF, TNF-α, and IL-10 secreted by activated NK cells, which is an important mechanism of NK cells to modulate adaptive immunity (34–36). When activated by activating signals, NK cells exert important regulatory functions in adaptive immunity, impacting both B cell and T cell responses (37).

It is crucial to have an understanding of the intricate regulatory role of NK cells in adaptive immune processes within various immune microenvironments. Such understanding aids in selecting optimal immunotherapeutic strategies. Our review comprehensively elucidates the regulatory role of NK cells in adaptive immune processes. By elucidating these regulatory mechanisms, we can gain insights into the intricate behavior of NK cells within distinct immune contexts, contributing to selecting the most effective immunotherapeutic approaches.

## 2 NK cells enter their site of action through various mechanisms

**Figure 1:**
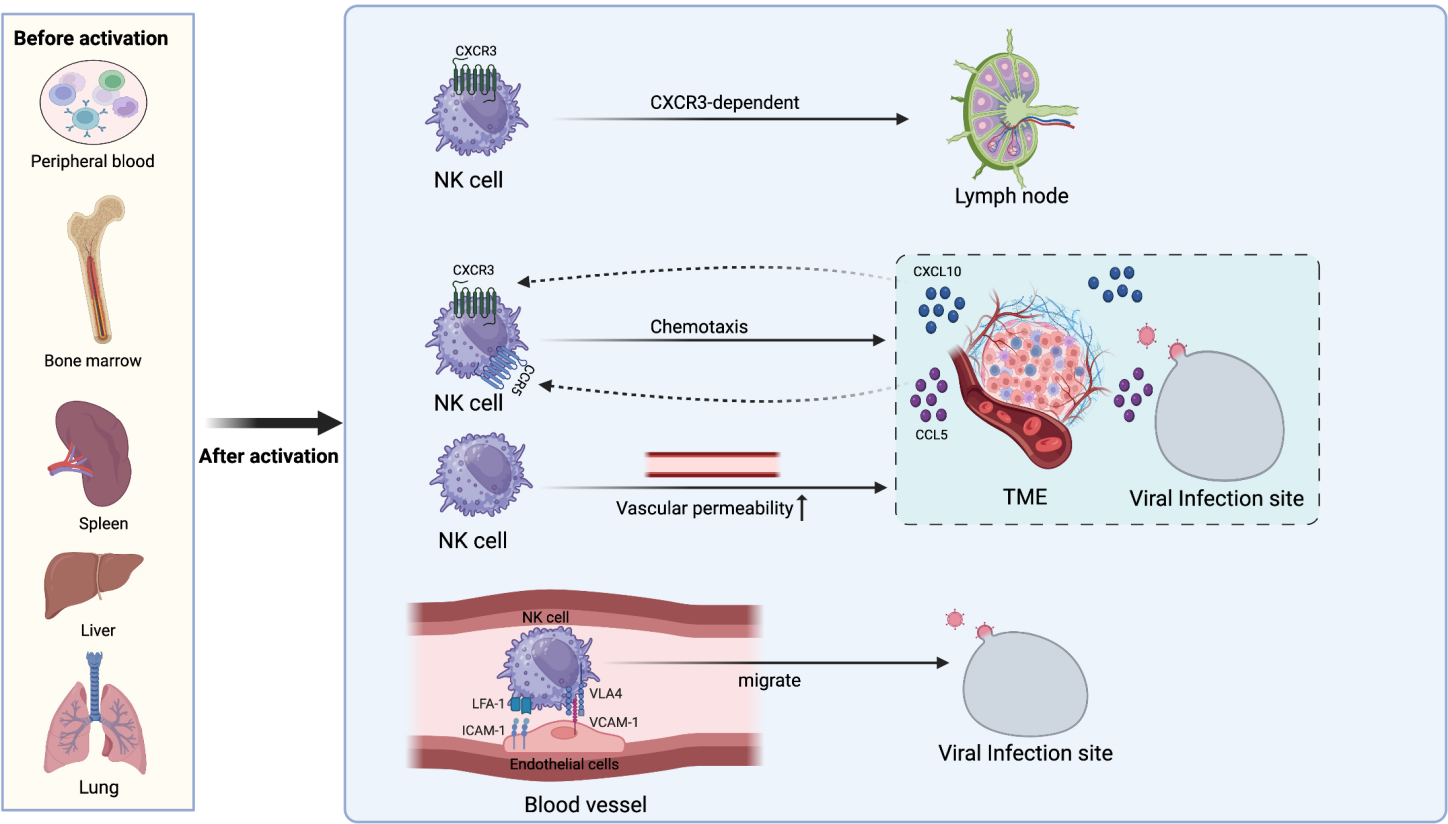
The localization and migration mechanism of NK cells. Before activation, NK cells are distributed in various locations such as the bone marrow, spleen, liver, lungs, and peripheral blood. After activation, NK cells can migrate to other sites through various means. They enter LN in a CXCR3-dependent manner to provide IFN-γ for TH1 priming. CXCL10 and CCL5 released from the TME and sites of viral infection drive NK cell chemotaxis to corresponding locations. Increased vascular permeability also facilitates NK cell extravasation from blood vessels to the TME and sites of inflammation. NK cells interact with endothelial cells at the infection sites through adhesion molecules such as LFA-1 with ICAM-1 and VLA-4 with VCAM-1, allowing them to roll along the vessel lumen and migrate towards the infection sites.

While NK cells are primarily distributed in peripheral blood, BM, spleen, lung, and liver before activation, they exhibit rapid responsiveness to infected or abnormal cells whenever required (38, 39). NK cells are chemotactically attracted to LN by chemokine during infection because of the expression of CXCR3. After entering the LN, NK cells furnish the surrounding T cells with the IFN-γ signals needed for their initial activation (40). Infection sites or tumor microenvironments can generate chemotactic factors such as CXCL10 or CCL5, which lead NK cells to migrate to these locations (41–43). Cell adhesion molecules are also crucial for the migration of NK cells towards infection sites. NK cells interact with the endothelial cells at the infection site through adhesion molecules, including the binding of VLA-4 to VCAM-1 and LFA-1 to ICAM-1 (44, 45). This supports the NK cells in rolling, adhering, and transmigrating across the vascular wall and into the site of infection. Moreover, tumor cells and inflammatory reactions can increase vascular permeability, enabling the passage of NK cells through the blood vessels to reach sites of inflammation or tumors (46). These studies demonstrate that NK cells can accumulate at sites of abnormalities in the body through various mechanisms, exerting their cytotoxic or regulatory functions (**Figure 1**).

## 3 Enhancement of T cell responses by NK cells

### 3.1 Cytokines and co-stimulatory molecules expressed by NK cells enhance T cell responses

Actually, NK cells mount an early antiviral response against cytomegalovirus infection while also modulating the intensity of adaptive immune responses. The response of CD8^+^T cells against the virus is significantly enhanced because NK cells contribute to regulation by producing optimal levels of IFN-α/β (47).

The TNF-α and IFN-γ secreted by NK cells that have been activated produce a stimulating response on the activation and proliferation of T cells (48). The IFN-γ released by NK cells can bind to the IFN-γR on the surface of naïve CD4^+^ T cells, initiating the transcription of T-bet (49). Consequently, naïve CD4^+^ T cells exhibit a tendency to differentiate into Th1 cells (40, 50), which ultimately enhances cell-mediated immune responses against infections.

**Figure 2:**
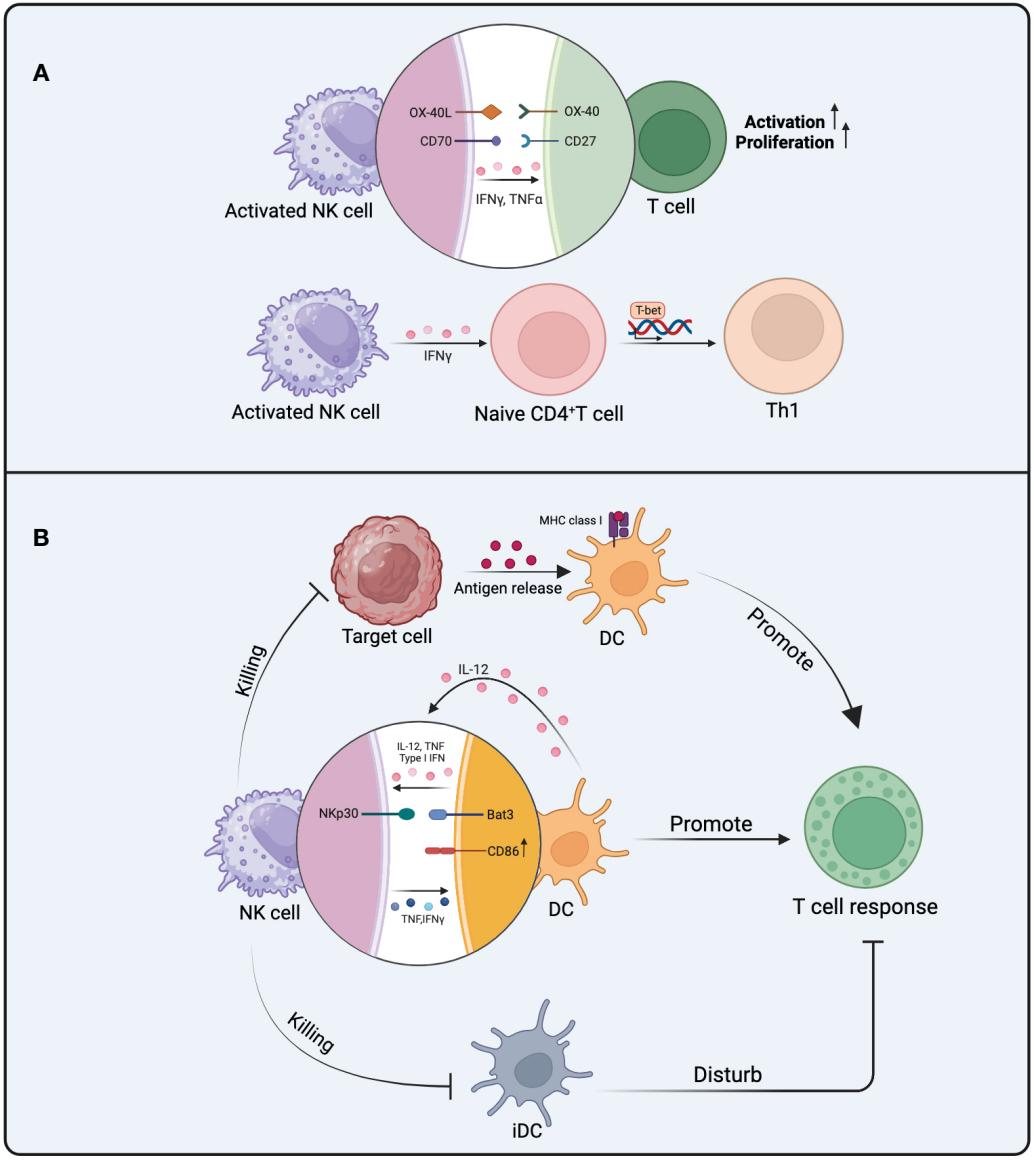
The mechanism of NK cells in promoting T cell responses. **(A)** The activation and proliferation of T cells influenced by activated NK cells involve the effects of OX40L interacting with OX40 and CD70 interacting with CD27. TNF-α and IFN-γ from NK cells also promote the activation and proliferation of T cells. The polarization of Th1 cells is further facilitated by IFN-γ from NK cells. **(B)** NK cells can impact T cell responses in three ways. Firstly, NK cell-mediated killing of target cells promotes antigen presentation by DCs, ultimately enhancing T cell responses. Secondly, there is mutual activation between DCs and NK cells, involving the participation of IL-12, TNF, and IFN. The binding of NKp30 to Bat3 provides additional activation signals to DCs, leading to an increase in the expression of CD86 in activated DCs. These favorable behaviors toward DCs promote T cell responses. Thirdly, the elimination of iDCs by NK cells reduces their interference with T cell responses.

In addition to enhancing T cell immune responses through the secretion of cytokines like TNF-α and IFN-γ, NK cells also impact T cell immune reactions by expressing specific co-stimulatory molecules. These molecules offer proliferation and activation signals to T cells. For example, the interaction between OX40 ligand expressed on NK cells and the OX40 receptor present on T cells efficiently delivers proliferation cues to the T cells (51). Although the expression of CD70 on NK cells is transient and tightly regulated, its interaction with CD27 on T cells can provide co-stimulatory signals, thereby promoting T cell survival and proliferation, leading to more efficient immune responses (52, 53) (**Figure 2A**).

### 3.2 NK cells enhance T cell response by improving DC maturation and activation

While there is direct crosstalk between T cells and NK cells, the regulation of T cell responses by NK cells is mainly achieved indirectly through the modulation of antigen-presenting cells (APCs) during the initial priming phase of T cell responses. APCs are crucial for T cell responses, as they not only present antigens to T cells but also provide the first and second signals for T cell activation (54). Dendritic cells (DCs), as a type of APC, undergo changes in their quantity, status, and functionality, all of which can influence T cell responses (55, 56).

Under different conditions, NK cells can influence DCs in various ways, subsequently impacting T cell response. Mature DCs (mDCs) migrate to LN in a CCR7-dependent manner and exhibit highly efficient stimulatory capabilities towards naïve T cells, whereas immature DCs (iDCs) persist in a stable state in the periphery (57). The mutual activation of NK cells and DC cells occurs during the initial stages of the immune response after they congregate in LN. Multiple investigations have shown that DCs enhance NK cell activity by releasing type I IFN, TNF,and IL-12 (57, 58). Conversely, activated NK cells secrete TNF and IFN-γ, influencing the maturation of DCs. DCs upregulate the expression of co-stimulatory molecules under the influence of TNF, and both TNF and IFN-γ synergistically improve DCs’ capability in generating IL-12 (59, 60). *In vitro* co-culture of iDCs with NK cells induces the maturation of DCs and enhances IL-12 secretion by DCs. The maturation process of DCs is strongly relies on cell-to-cell contact with NK cells, even though the generation of TNF and IFN-γ by NK cells is already crucial for DC maturation (57). *In vivo* imaging techniques have revealed direct contacts between NK cells and DCs (61). IFN-γ from NK cells not only stimulates the maturation of DCs but also heightens the expression of MHC-I on DCs (62). NK cells must possess a specific inhibitory receptor that permits them to discern MHC class I molecules (63). Once the expression of MHC class I molecules on DCs is upregulated, it reduces the likelihood of their recognition as non-self cells by NK cells, thereby establishing stronger immune tolerance (64). This suggests that IFN-γ participates in promoting the maturation of DCs and providing them with enhanced protection, enabling them to assume a more significant role in promoting T cell responses. The addition of lipopolysaccharides (LPS) into the co-culture system amplifies the NK cells’ ability to enhance DC maturation. This results in a notable increase in the expression of the co-stimulatory molecule CD86 on DC surfaces and a heightened release of IL-12 (65). Following this, after being stimulated by CD40L, DCs produce an increased amount of IL-12p70, consequently bolstering the T cell response (66). Despite IL-2-activated NK cells can also trigger DC maturation and bolster their capacity to activate naïve CD4^+^ T cells from the same species but different donors. Nevertheless, when iDCs are co-cultured with NK cells in the presence of IL-2, NK cells exhibit cytotoxicity towards iDCs and secrete IFN-γ (67). Interactions between the NKp30 receptor found on NK cells and Bat3 expressed on DCs have been observed, contributing to the activation and maturation of DCs. It can provide additional signals for inducing DC maturation while recognizing DCs (68).

The “DC editing” process of NK cells is a crucial pathway through which NK cells promote T cell immunity. Since the antigen-presenting ability is typically found in mDCs rather than iDCs, NK cells selectively eliminate iDCs and spare the survival of immunogenic mDCs, thus facilitating effective T cell immune responses. mDCs are not susceptible to lysis by NKG2A^+^ NK cells due to their higher expression levels of HLA class I molecules compared to iDCs. Furthermore, as the levels of NKG2A on NK cells decrease, their sensitivity to the HLA-E expression of iDCs increases (69). NKp30 significantly contributes to the “DC editing” process by recognizing and eliminating iDCs (70), and this process relies on MHC-I expression, as mDCs exhibit higher levels of MHC-I, allowing them to escape NK cell recognition (71). Similar phenomena have been observed in tumor immunology research, wherein the elimination of iDCs by NK cells is pivotal for the proliferation of tumor-specific CTLs (Cytotoxic T Lymphocytes) (72).It is intriguing that, under inflammatory conditions, iDCs are also susceptible to NK cell cytotoxicity. This phenomenon can be comprehended as a “braking mechanism” that occurs after the resolution of the inflammatory response. It serves to regulate the quantity of DCs capable of initiating T cell responses, thereby preventing the development of excessive inflammatory reactions (73). Additionally, in studies focusing on mouse DC vaccines, NK cells enhance antigen-specific T cell responses by killing iDCs through the TRAIL pathway (74). However, further validation is required to ascertain whether these iDCs obtained through *in vitro* culture represent the *in vivo* state accurately (69) (**Figure 2B**).

### 3.3 NK cells augment antigen presentation opportunities for DCs

The existing evidence indicates NK cells have significant impact on the cross-presentation ability of DCs. One perspective is DCs can uptake antigens which from target cells killed by NK cells and present them via MHC-I molecules (75, 76). This process enhances antigen presentation, leading to effective activation of adaptive immune responses. When allogeneic B cells are transplanted into mice, NK cells mediate the identification and eradication of non-self cells, thereby facilitating DC phagocytosis of apoptotic bodies and the antigen presentation process. This initiates subsequent adaptive immune responses (76). *In vivo*, the lysis of OVA-expressing splenocytes mediated by NK cells leads to antigen release, which is taken up by DCs and enables efficient activation of CD8^+^ T cell and CD4^+^ T cell responses (75). Evidently, the ability of NK cells to lyse abnormal cells may facilitate DCs in capturing and cross-presenting antigens, ultimately promoting adaptive immunity.

On the other hand, the antigen presentation process by DCs requires assistance from NK cells. The antigen presentation by DCs may become compromised without this supportive role. *In vitro* experiments concerning the antigen presentation from DCs to CD8^+^ T cells, the necessity of this assisting role of NK cells has been established. This is because the maturation of DCs and the capture of tumor cells are closely intertwined with the involvement of NK cells (77). It has been confirmed that depleting NK cells in a murine melanoma model results in the complete loss of CD8^+^ T cell response initiation. This is because the stimulation of CD8^+^ T cells through antigen presentation is heavily reliant on the presence of NK cells (78). Also, in an experiment using monocytes derived from PBMCs and induced to a semi-mature phenotype of DCs through IFN-α and GM-CSF, the presence of NK cells is necessary under these culture conditions, as DCs cannot effectively stimulate T cell immunity without NK cells (79). Upon stimulation by NK cell-secreted IFN-γ and TNF, these monocyte-derived DCs efficiently present tumor-derived antigens, thereby promoting the activation of tumor-reactive CD8^+^ T cell responses (80). In summary, both the antigen cross-presentation capacity and antigen load of DCs are regulated by NK cells. These influences have profound implications for subsequent T cell responses (**Figure 2B**).

## 4 Inhibition of T cell responses by NK cells

### 4.1 Cytokine-mediated suppression of T cell function by NK cells

In preceding scientific inquiries, the negative regulatory function of NK cells in adaptive immunity has not been as extensively studied as their promoting effect on adaptive immunity. However, similar to regulatory T cells (Tregs) exerting regulatory functions by suppressing the activity of other immune cells, the concept of regulatory NK cells has been proposed early on (81). This implies that T cell responses can be negatively regulated by NK cells.

**Figure 3:**
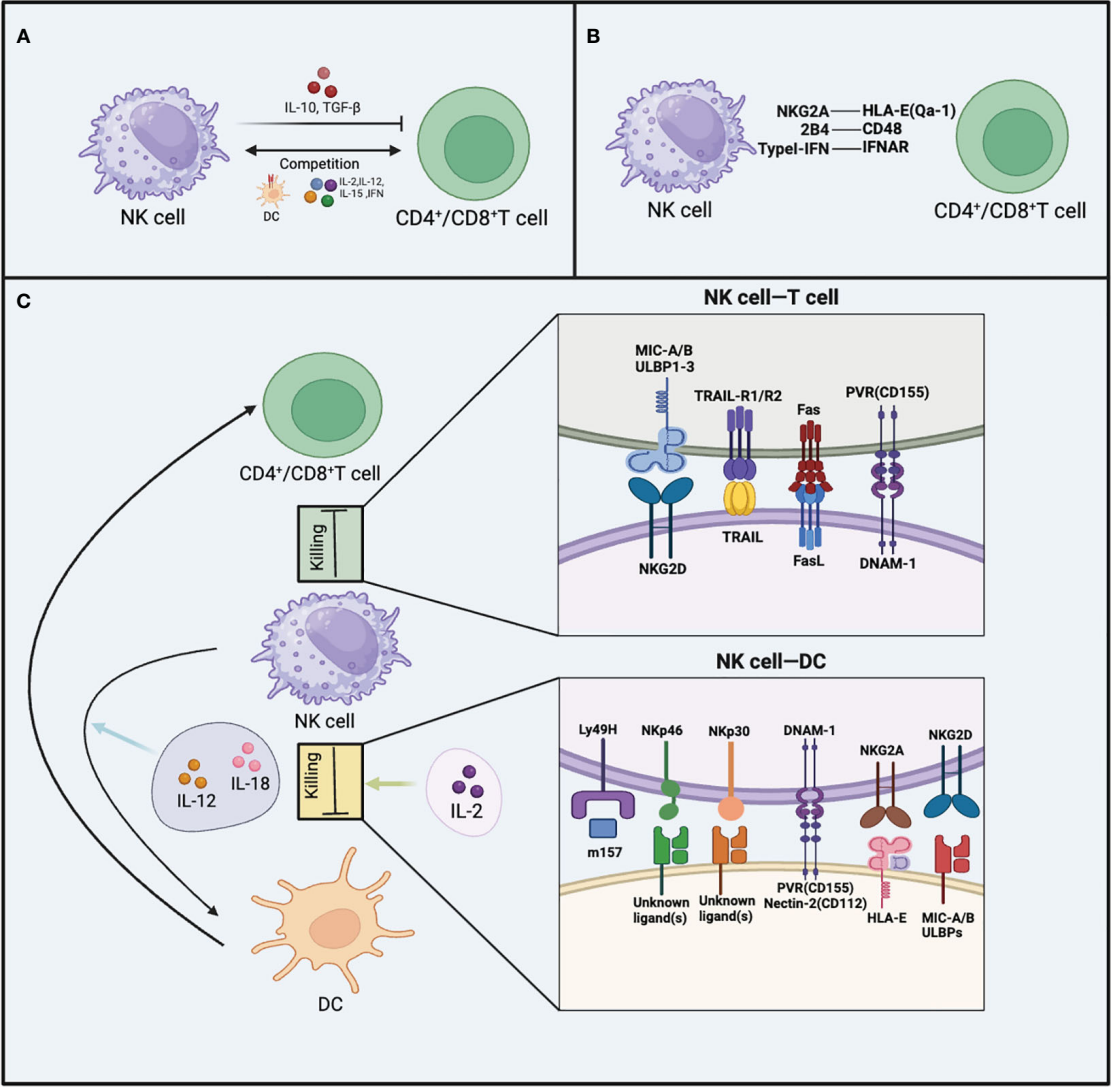
The inhibitory effects of NK cells on T cells and DCs. **(A)** The production of TGF-β and IL-10, as well as competing with CD4^+^ T cells and CD8^+^ T cells for cytokines like TNF, IL-15, IL-12, IL-2, and MHC-I on DCs, are all mechanisms by which NK cells inhibit T cells. **(B)** The figure illustrates three receptor-ligand interactions that enable CD4^+^ T cells and CD8^+^ T cells to evade targeting by NK cells, including NKG2A-HLA-E (Qa-1), 2B4-CD48, and Type I IFN-IFNAR. **(C)** NK cells employ various receptor-ligand interactions to target CD4^+^/CD8^+^ T cells, as well as DCs. For CD4^+^/CD8^+^ T cells, these interactions involve TRAIL, NKG2D, FAS/FASL, and DNAM-1. Regarding DCs, the receptor-ligand interactions include Ly49H, NKp46, NKp30, DNAM-1, NKG2A, and NKG2D.NK cells exhibit different interactions with DCs when stimulated by various cytokines.

TGF-β and IL-10 are cytokines secreted by NK cells that exert inhibitory effects on T cell function (82, 83). NK cells can secrete IL-10 during viral infections and indirectly affect T cells by non-contact-dependent mechanisms, inhibiting antigen-specific T cell proliferation (84–86). During the course of lymphocytic choriomeningitis virus (LCMV) infection, NK cells can suppress CD8^+^ T cell responses by secreting IL-10, thus causing suboptimal control of viral infection (87). When perforin-deficient mice are infected with MCMV, the viral infection persists, and NK cells secrete IL-10 to inhibit the function of CD8^+^ T cells (88). In human PBMCs, IL-10-secreting NK cells have been observed, and they can inhibit antigen-specific CD4^+^ T cell proliferation *in vitro* (89). Surprisingly, the knockout of the IL-10 gene in NK cells did not lead to an enhancement of T cell responses during chronic LCMV infection (90), casting uncertainty on the functional relevance of IL-10 derived from NK cells in the context of viral infections. This implies that the mechanism by which NK cells suppress T cell function is not singular (**Figure 3A**).

### 4.2 Competition between T cells and NK cells limits T cell function

The phenomenon of competitive inhibition is evident between T cells and NK cells, whereby T cell functionality is likewise suppressed as a result of such interactions. NK cells can engage in competition with CD4^+^ T cells for binding to MHC-II on DCs, resulting in a reduction of accessible antigen signals for CD4^+^ T cells. Consequently, this limited availability of antigen signals restricts the proliferation of CD4^+^ T cells (91). There is also competition between NK cells and CD8^+^ T cells. Using TGF-β antibody and IL-2 for combined anti-tumor therapy, there is dual competition between activated NK cells and CD8^+^ T cells. After combination treatment, depletion of either activated CD8^+^ T cells or NK cells leads to the expansion and compensatory anti-tumor effects of the remaining cell population (92). T cells and NK cells also compete in their interaction with cytokines, as both express various identical cytokine receptors, including IFN-γ, IFN-α/β, IL-15, IL-12, and IL-2 (93–96). During MCMV infection, there exists a distinctive phenomenon whereby NK cells rapidly upregulate IL2Rα (CD25) (97). This upregulation allows NK cells to bind more IL-2, promoting their own proliferation. This observation also implies the likelihood of T cells and NK cells competing for IL-2 binding. Additionally, the homeostatic proliferation of CD8^+^ T cells is constrained by NK cells, but this restraint can be overcome by supplementing IL-15 (98). This indicates potential competition between CD8^+^ T cells and NK cells for limited levels of IL-15 (**Figure 3A**).

### 4.3 T cell responses suppressed by direct killing from NK cells

NK cells exert their most direct inhibitory effect on T cells through direct killing. Both human and murine experiments have manifested that T cells can be directly killed by NK cells. During the development of chronic colitis, NK cells alleviate the immunopathological condition by exerting cytotoxic effects on effector CD4^+^ T cells (99). It’s worth noting that most T cells sensitive to NK cell cytotoxicity are in an activated state, while resting T cells exhibit a degree of resistance to NK cell-mediated killing (100, 101). The window of sensitivity of activated T cells to NK cell cytotoxicity is also primarily within the early stages of their activation. As time progresses, the sensitivity decreases until the T cells encounter the same antigen again. The upregulation of NKG2D ligands on activated T cells may be the reason for their increased sensitivity to NK cell cytotoxicity (102, 103). ULBP1, ULBP2, ULBP3, and MICA are NKG2D ligands that activated CD8^+^ T cells and CD4^+^ T cells may upregulate, and NK cells use NKG2D to recognize whether these T cells are in an activated state (104). Following autologous IL-2 activation of NK cells, activated CD8^+^ T cells and CD4^+^ T cells are killed by NK cells through a perforin-dependent mechanism (104). Upon NK cell depletion, the number of memory CD8^+^ T cells significantly increases after vaccination, suggesting that NK cells may have a direct cytotoxic effect on T cells. This cytotoxic effect also relies on the release of perforin and the expression of NKG2D (103). While NK cells’ inhibitory effects on T follicular helper (Tfh) and B cells also involve the release of perforin, NKG2D is not implicated in these processes (105). TRAIL is expressed by NK cells, and activated T cells express TRAIL receptors, including TRAIL-R1 (DR4) and TRAIL-R2 (DR5).There have been reports indicating that NK cell-mediated killing of CD4^+^ T cells involves TRAIL molecules (101). CD56^bright^ NK cells expressing TRAIL in humans can selectively bind to TRAIL receptors on activated T cells, thereby inducing T cell apoptosis (101). After stimulation by superantigens and during the proliferative phase (S phase, G2M phase), T cells upregulate DNAM-1 ligands such as PVR. NK cells preferentially kill proliferating T cells through the activation receptor DNAM-1 (106). Tregs, like other T cells, are also subject to the cytotoxic effects of NK cells. Activated human NK cells can kill Tregs during the cellular antigen response, and the elimination of Tregs by NK cells may be a mechanism by which NK cells promote T cell responses (107). In addition, NK cell-mediated cytotoxicity against T cells also involves the Fas/FasL pathway (108).

In both chronic and acute LCMV infections, NK cells can eliminate T cells activated *in vitro*. The presence of NK cells accelerates the viral infection process because the depletion of NK cells increases the frequency of LCMV-specific CD8^+^ T cells and reduces viral titers (102). However, it should not be overlooked that the role of NK cells during LCMV infection is also dependent on the infecting dose and the strain of the virus. LCMV-Clone13 strain establishes widespread chronic infection in the host, while LCMV-Armstrong strain causes acute infection. In high-dose LCMV-Clone13 virus infection, the presence of NK cells prevents mouse death but leads to persistent viral infection. Although the depletion of NK cell population increases the number of T cells and improves T cell exhaustion, the survival of mice is not optimistic. In moderate-dose LCMV-Clone13 virus infection, NK cell deplete completely eliminates viral infection and blocks lethal immune-mediated pathological processes (109). In contrast to the previously described mechanisms, in this study, the inhibition of CD8^+^ T cells by NK cells is an indirect consequence of NK cell-mediated suppression of CD4^+^ T cells, rather than a direct interaction between CD8^+^ T cells and NK cells (109). When mice are infected with low doses of LCMV-Clone13 or LCMV-Armstrong strains, the depletion of NK cells only has a weak effect on T cell responses, and mild tissue pathology is present in the organs regardless of NK cell depletion (102, 109, 110) (**Figure 3C**).

### 4.4 The behavior of T cells evading NK cell-mediated cytotoxicity

Both non-classical and classical MHC-I molecules expressed on autologous cells are crucial for evading NK cell-mediated killing (111). Classical MHC-I molecules are expressed on self-nucleated cells and interact with inhibitory receptors including KIR in humans or Ly49A, C, and D receptors in mice (18). The expression of MHC-I molecules is regulated by NLCR5, and T cells lacking NLCR5 become susceptible to NK cell targeting (112). Mouse Qa-1 and human HLA-E, which belongs Non-classical MHC-I molecules have the capability to interact with NKG2A, thereby preventing the lysis of self-cells by NK cells (113). Under normal conditions, the downregulation of MHC-I molecule expression occurs in infected cells or tumor cells, rendering these cells sensitive to NK cell-mediated killing (114). Activated T cells need to modify their state to avoid being recognized by NK cells. Activated CD8^+^ T cells and CD4^+^ T cells lacking type I interferon receptors can be targeted by NK cells, leading to the secretion of perforin and killing of these cells during the acute LCMV infection phase (115). T cells expressing type I interferon receptors also avoid recognition and killing by NK cells through the absence of NKp46 ligands (115). Researchers were surprised to discover that when NK cell-regulated T cell responses during LCMV viral infection, NK cells lacking the 2B4 receptor can cause cytotoxicity in activated CD8^+^ T cells, even when these T cells express MHC-I molecules (116). This cytotoxic behavior of NK cells is alleviated when the 2B4 receptor is expressed or when NK cells are depleted, suggesting the involvement of the 2B4 receptor in the evasion of LCMV-specific CD8^+^ T cell killing by NK cells (117). In summary, the expression of ligands for inhibitory receptors and MHC molecules by T cells can both effectively help T cells evade NK cell-mediated cytotoxicity (**Figure 3B**).

### 4.5 APCs modulation by NK cells influences T cell immune response

The changes in APCs induced by NK cells not only exert promoting effects on T cell responses but also demonstrate inhibitory effects. In the context of MCMV infection, the depletion of NK cells leads to an amplified proliferative ability and elevated IFN-γ production by T cells (110). Subsequent studies revealed that factors restricting T cell antiviral responses and survival are attributed to the targeting of MCMV-infected DCs by NK cells, thereby weakening the antigen sensing by T cells (118). NK cells directly recognize and eliminate DCs presenting the m157 protein through Ly49H, leading to a reduced number of antigen-presenting DCs during MCMV infection. Consequently, this impairment of DC antigen presentation results in compromised T cell immunity. However, if Ly49H^+^ NK cell-mediated killing of DCs is inhibited, the T cell immune response in mice can be restored (118). In the LCMV model, a similar phenomenon exists. In the absence of NK cells, APCs stimulate CD8^+^ T cell activation and enhance CD8^+^ T cell cytotoxicity, leading to effective control of viral infection. However, NK cells need to be eliminated within the first two days of viral infection. This enhancement is attributed to the increased number of APCs after the removal of NK cells, rather than an augmentation of APC co-stimulatory capacity (119).Additionally, NK cell-mediated elimination of DC populations may also involve the activating receptor NKp46, as mutations in the NKp46 gene have been reported to cause excessive NK cell responses and failure to mount optimal anti-MCMV responses (120). NKG2A also participates in the killing of DCs, iDCs express lower levels of HLA-E, making them a primary target for NK cells via NKG2A-mediated cytotoxicity. However, even mDCs expressing high levels of HLA-E can still be partially targeted and eliminated by certain NK cells (69). The receptors DNAM-1 and NKp30 on NK cells may synergistically contribute to the elimination of DC populations. The impact of DNAM-1 receptor on DC killing partially depends on the expression levels of Nectin-2 and PVR on DCs (121). It is worth noting that during chronic viral infections, NK cells can produce IL-10, which induces distinct phenotypic changes in DCs. Under the influence of IL-10, iDCs upregulate MHC-I expression, while mDCs downregulate MHC-I expression. Subsequently, NK cells promote upregulation of activating receptor NKG2D expression, contributing to the recognition and elimination of mDCs through NKG2D-mediated cytotoxicity. This circumstance inevitably leads to the accumulation of numerous immunogenically poor iDCs in the LN, ultimately resulting in impaired immune function (122).

The interaction between DC populations and NK cells depends on various factors. Whether their interaction promotes maturation or leads to cytotoxicity depends on an important factor, which is the ratio of DCs to NK cells. At a low NK : DC ratio (1:5), NK cells promote DC expansion and their ability to secrete cytokines. On the other hand, at a high NK : DC ratio (5:1), NK cells mediate cytotoxicity against autologous DCs (65). Accordingly, changes in the ratio between NK cells and DCs in different disease environments can affect subsequent T cell response.

In fact, the impact of NK cells on DCs is further influenced by the kind of cytokine stimulation received by NK cells. For instance, under the stimulation of IL-2, NK cells display a cytotoxic state towards DCs. Moreover, NK cells can promote DCs to release IL-12 when activated by IL-18 (123). IL-18-activated NK cells also secrete a highly migratory and pro-inflammatory molecule, HMGB1(high-mobility group box 1), which aids in protecting DCs from NK cell-mediated killing and promotes DC maturation (124). NK cells activated by IL-12 have also been shown to promote DC maturation and enhance their capacity to induce Th1 cell production of IFN-γ (125). Therefore, the response of NK cells to different cytokine stimulations results in distinct behaviors of DCs, leading to diverse immune responses (**Figure 3C**).

Furthermore, due to the activation of NK cells by low doses of IL-15 delivered in a trans-presentation manner through APCs (126), IL-15 exerts a more intricate influence on NK cell immune modulation. IL-15 can promote myeloid DCs to produce IL-12, which subsequently acts on NK cells and impacts their activation (127). While IL-12 is not a decisive factor for NK cell activation, it effectively enhances NK cell secretion of IFN-γ (128). NK cells lacking IL-18 signaling fail to secrete IFN-γ when stimulated with IL-12 *in vitro*, but the induction of IFN-γ transcription levels by IL-12 is similar in NK cells with or without intact IL-18 signaling. This suggests that IL-18 stimulation of NK cells may improve the translation of IFN-γ mRNA (129). The actions of IL-12, IL-15, and IL-18 on NK cell status and function are complex, indicating that various cytokines can influence NK cell regulatory behavior. Furthermore, the synergistic effects of multiple cytokines or the absence of specific cytokine actions may introduce novel influences on NK cell immune modulation.

## 5 Dual regulation of B cell responses by NK cells

### 5.1 Promotion of B Cell responses by NK cells

Direct or indirect communication between B cells and NK cells gives rise to various regulatory influences of NK cells on B cell responses.

**Figure 4:**
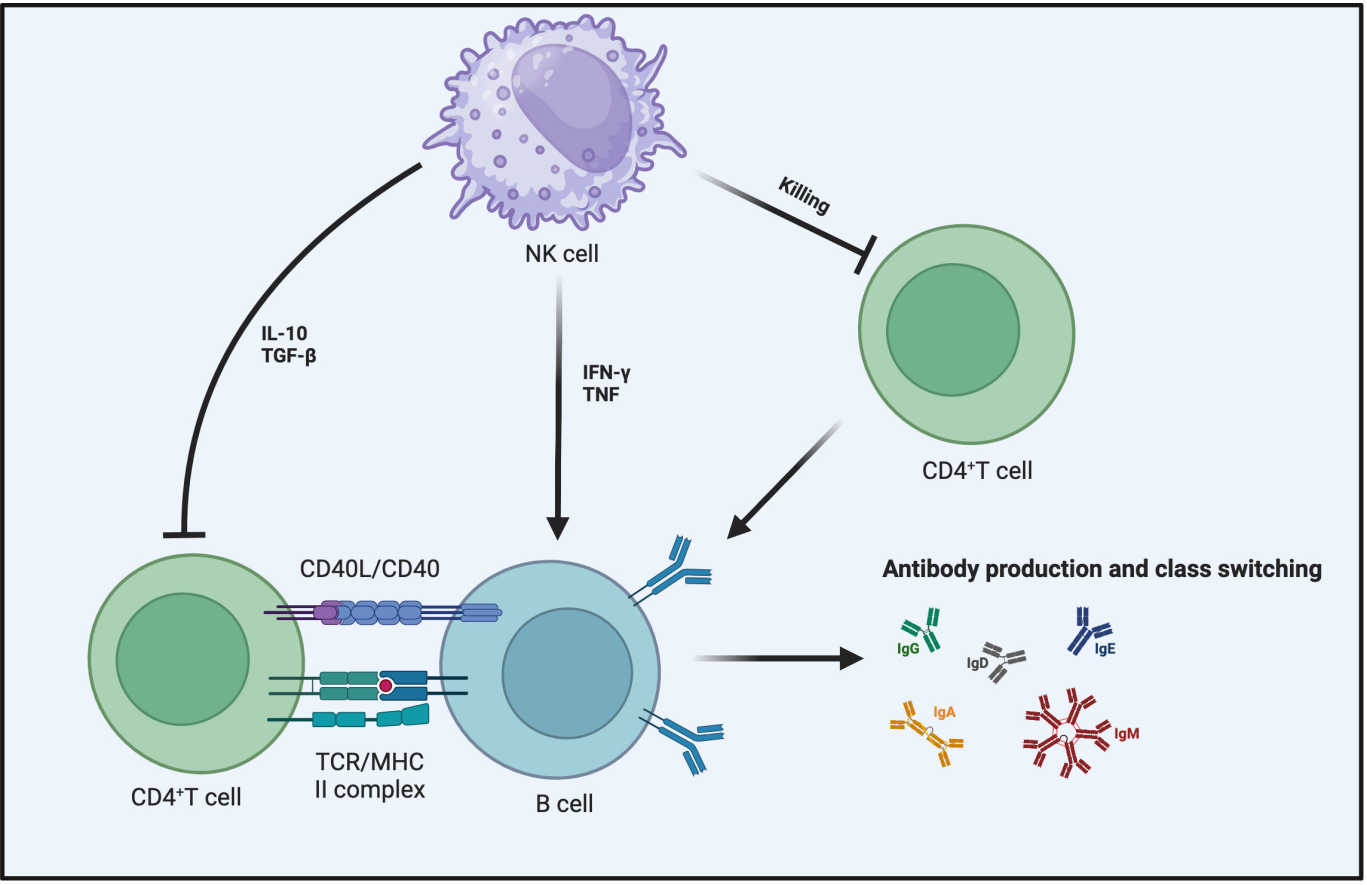
NK cells impact B cell responses from multiple angles. NK cells can secrete IFN-γ and TNF to promote B cell maturation and enhance their ability to produce antibodies and undergo antibody class switching. In the context of inhibiting B cell responses, aside from the direct killing of B cells by NK cells, the primary mechanisms involve the influence of NK cells on the interaction between CD4^+^ T cells and B cells. This includes the role of TGF-β and IL-10 in suppressing CD4^+^ T cell responses or making CD4^+^ T cells more susceptible to NK cell-mediated killing.

The influence of activated NK cells on B cell responses is multifaceted, in certain instances, activated NK cells can enhance B cell responses (130). *In vitro* co-culturing of B cells with NK cells results in NK cells enhancing B cell activation, promoting immunoglobulin (Ig) production, and facilitating antibody class switching (131–133). Although these effects may involve IFN-γ, TNF, and CD40-CD40L interaction, they are independent of T cells (134–136). The proliferation capacity and subtype switching of B cells are influenced by NK cell-secreted IFN-γ, as the presence of IFN-γ leads to alterations in B cell proliferation capacity and subtypes (137). Similarly, NK cells have the potential to promote antibody production by B cells and activate B cells *in vivo (* 138). In experiments where mice lacking NK cells were immunized with ovalbumin or keyhole limpet hemocyanin, a reduction in antigen-specific IgG2a production occurred when certain NK cell stimulants, such as polyI:C or complete Freund’s adjuvant, were used (139–142). This highlights the indispensable role of NK cells in the *in vivo* environment for B cells (**Figure 4**).

### 5.2 NK cells employ various mechanisms to inhibit B cell responses

Contrary to the aforementioned studies highlighting NK cell-enhanced B cell responses, several reports have described in detail the inhibitory role of NK cells in humoral immunity during viral infections and vaccination processes. In research carried out with human subjects, NK cells can inhibit B cell proliferation and prevent their differentiation into plasma cells (143, 144). Under many conditions, both murine and human NK cells can kill activated B cells (145–149). Multiple *in vitro* studies have indicated that NK cells can inhibit B cell antibody responses triggered by mitogenic stimulation with phytohemagglutinin (150). Since the sensitivity of B cells to natural killer activity is related to their cell differentiation status, the killing effect of NK cells on B cells appears to be selective. Using B cells at different stages as target cells for NK cell killing demonstrates that B cells in the late stage are more susceptible to recognition and killing by NK cells (151).

The inhibitory effect of NK cells on T cell responses ultimately affects B cell reactions as well. TGF-β and IL-10 expressed by NK cells can indirectly inhibit B cell responses by suppressing T cell reactions (86, 152, 153). NK cells achieve the regulation of B cell responses indirectly by suppressing helper CD4^+^ T cells (154–156). This inhibitory mechanism of NK cells may involve direct cell-cell contact or cytokine secretion. NK cell inhibition of Tfh responses limits humoral immunity during chronic and acute viral infections (105, 157). Therefore, when NK cells are depleted, the antibody response becomes more sustained because the reduction in the numbers of Tfh cells and GC B cells is alleviated (105). Depleting NK cells before LCMV infection in mice leads to abundant Tfh cells, promoting the formation of germinal centers and plasma cells, then enhancing control of the viral infection and increasing the concentration of anti-LCMV antibodies (157). The inhibitory effect of NK cells on Tfh responses also occurs during the immunization process of vaccines, consequently constraining the vaccine-induced germinal center-mediated antibody affinity maturation (158). Disrupting this mechanism during HIV infection also contributes to the generation of high-affinity broadly neutralizing antibodies (159). Surprisingly, the killing effect of activated NK cells on T cells is also influenced by the inhibitory receptor-ligand expressed on B cells (160). All of the aforementioned observations also highlight that while B cell responses and T cell responses are distinct, they do exhibit intersecting regulatory mechanisms within the immune system (**Figure 4**).

## 6 Conclusions

Initially, NK cells were regarded as the “innate guardians”. Indeed, NK cells live up to expectations by protecting the body against foreign attacks in many cases. However, with further research, the “dark” side of NK cells has gradually been revealed. It is recognized that not everything is beneficial and harmless in all circumstances, and the same applies to NK cells. NK cells regulate adaptive immunity, and this regulation can occur through multiple pathways, both promoting and inhibiting the processes of adaptive immunity. The role of NK cells in viral infections as well as tumor immune processes also vary with changing conditions. Although the above discussion focuses on a wide range of immune regulation mediated by NK cells, we still have limited knowledge of how NK cells will function under different conditions and undergo different processing. Why do NK cells exhibit cytotoxicity towards T cells or B cells? The Intracellular mechanisms underlying their cytotoxic actions lack a clear understanding. Hence, elucidating the heterogeneity of NK cells under diverse conditions holds paramount significance. This also highlights the importance of carefully selecting therapeutic agents in NK cell-based immunotherapy and evaluating whether the functionality and state of NK cells may be altered during treatment or research. It is crucial to prevent the dominant suppression of immune responses by NK cells.

## Author contributions

HJ: Conceptualization, Investigation, Methodology, Supervision, Writing – original draft, Writing – review & editing. JJ: Conceptualization, Funding acquisition, Investigation, Methodology, Resources, Supervision, Writing – original draft, Writing – review & editing.
